# Temporal trends in Tdap vaccination coverage during pregnancy in the Vaccine Safety Datalink, 2016 through 2023

**DOI:** 10.1016/j.vaccine.2026.129175

**Published:** 2026-09-22

**Authors:** Stephanie A. Irving, Bradley Crane, Eric S. Weintraub, Hilda Razzaghi, Mehreen Meghani, Carla Black, Sarah C.J. Jorgensen, Matthew F. Daley, Candace C. Fuller, Sharon Fuller, Darios Getahun, Sungching C. Glenn, Simon J. Hambidge, Lisa A. Jackson, Elyse O. Kharbanda, Nicola P. Klein, Sean T. O’Leary, Teresa Schmidt, Katharine Raisler, Nancy S. Weinfield, Joshua T.B. Williams, Ousseny Zerbo, Allison L. Naleway

**Affiliations:** a Kaiser Permanente Center for Health Research, Portland, OR, USA; b Immunization Safety Office, Centers for Disease Control and Prevention, Atlanta, GA, USA; c Immunization Services Division, Centers for Disease Control and Prevention, Atlanta, GA, USA; d Marshfield Clinic Research Institute, Marshfield, WI, USA; e Institute for Health Research, Kaiser Permanente Colorado, Denver, CO, USA; f Harvard Pilgrim Health Care Institute and Harvard Medical School Boston, MA, USA; g Kaiser Permanente Washington Health Research Institute, Seattle, WA, USA; h Kaiser Permanente Southern California, Pasadena, CA, USA; i Denver Health, Denver, CO, USA; j Health Partners Institute, Minneapolis, MN, USA; k Vaccine Study Center, Kaiser Permanente Northern California, Oakland, CA, USA; l OCHIN, Portland, OR, USA; m University of Colorado Anschutz Medical Campus, Aurora, CO, USA; n Kaiser Permanente Mid-Atlantic Permanente Research Institute, Washington, DC, USA

**Keywords:** Tdap vaccine, Maternal vaccination, Pregnancy, Vaccination coverage, Vaccine uptake

## Abstract

**Background:**

In the United States (US), the tetanus, diphtheria, pertussis (Tdap) vaccination is recommended during every pregnancy. However, recent US survey data indicate uptake of the vaccine may be declining. We assessed Tdap vaccination coverage during pregnancy at ten health systems participating in the Vaccine Safety Datalink (VSD) to further examine recent trends.

**Methods:**

Using a retrospective cohort, we examined Tdap vaccination coverage among pregnant women aged 12–55 years with a live birth of ≥26 weeks’ gestation from January 2016 through December 2023; pregnancies were identified using a validated algorithm. We captured all Tdap, influenza and COVID-19 vaccination during the pregnancy using electronic health records linked to immunization registries. Among women with births in each calendar year, we assessed unadjusted Tdap vaccination coverage overall and by age group and race and ethnicity.

**Results:**

Between 102,374 and 112,297 pregnancies were included in coverage estimates each year. In each year, the population was 33–36% non-Hispanic (NH) White and 62–63% of mothers were 25–34 years old. Tdap vaccination coverage was 84.8% among those with births in 2016, peaked at 88.7% among those with births in 2019 and declined steadily to 83.9% among those with births in 2023. Coverage was consistently lower among those aged 12–17 and 18–24 years, compared to mothers aged ≥25 years. In all years, NH Asian mothers had the highest Tdap vaccination coverage and NH Black mothers had the lowest coverage, with differences of 11.5 to 15.8 percentage points between the two groups each year.

**Conclusion:**

Between 2016 and 2023, Tdap vaccines were administered in more than 80 % of pregnancies ending in live births in the VSD. Gains in coverage seen between 2016 and 2019 were offset by declines in later years. Despite the robust overall coverage, we identified consistent differences by age and race and ethnicity, with gaps widening in later years.

## 1. Introduction

Tetanus-diphtheria-acellular pertussis (Tdap) vaccination during pregnancy protects pregnant women and their infants [1,2]. Young infants are at greatest risk for hospitalization and death due to pertussis; more than 20% of infants <6 months of age with reported pertussis were hospitalized in 2023 [3], and infants <1-year-old account for 88% of pertussis deaths [2,4]. Because the multi-dose series diphtheria-tetanus-acellular pertussis (DTaP) vaccines are not recommended until two months of age, very young infants are dependent on passively acquired maternal antibodies [1,2]. Accordingly, Tdap vaccination is recommended during pregnancy. As of this writing, the United States Advisory Committee on Immunization Practices (ACIP) recommends Tdap vaccination in every pregnancy during gestational weeks 27 through 36, with a preference for the earlier part of that period [5].

In the United States, Tdap vaccines were first recommended during every pregnancy in October 2012 [6]. Vaccination coverage among pregnant women in the United States increased dramatically following the recommendation, with one national estimate finding more than 50% of privately insured pregnant women received Tdap in 2016 [7]. Coverage among publicly insured women did not increase at the same rate, with Medicaid data indicating only 23% of pregnant women received Tdap in 2016, increasing to 31% in 2019 [8]. More recently, a 2024 Centers for Disease Control and Prevention (CDC) Internet panel survey reported Tdap vaccination coverage of 60% among respondents with a recent live birth. The survey identified variable coverage by demographic characteristics, including race [9]. The 2025 Internet panel survey found an overall decrease in coverage, with 52.6% of pregnant women with Tdap receipt and statistically significant differences by race [10]. Ongoing monitoring of vaccination coverage is crucial to identify health care gaps and tailor vaccination strategies.

To complement existing coverage estimates, explore changes in Tdap uptake over time, and inform future vaccine safety research among pregnant women, we examined Tdap vaccination coverage by calendar year among pregnant women within the Vaccine Safety Datalink (VSD) with live births between January 2016 and December 2023.

## 2. Methods

### 2.1. Setting

The VSD is a long-standing collaborative effort between CDC’s Immunization Safety Office and 13 integrated health care systems (sites) across the United States [11,12]. VSD sites create standardized, de-identified data files from electronic health record (EHR) and insurance claims data, allowing linkage of select demographics, medical encounter information (including procedures and diagnoses), and vaccination history. Data for this analysis were obtained from ten VSD sites (Harvard Pilgrim Health Care Institute, HealthPartners, Marshfield Clinic, Denver Health, and the Colorado, Mid-Atlantic, Northern California, Northwest, Southern California, and Washington regions of Kaiser Permanente) in nine US states (California, Colorado, Massachusetts, Maryland, Minnesota, Oregon, Virginia, Washington, and Wisconsin) and Washington, D. C. All ten participating sites contributed data for the duration of the study period. Studies show that the demographic characteristics of individuals enrolled in these health care systems are broadly representative of the geographic areas covered by the health systems and the general US population [13,14].

This activity was reviewed and approved by the institutional review boards of the ten participating VSD sites with a waiver of informed consent. In addition, the activity was reviewed by CDC, deemed not human subjects research, and was conducted consistent with applicable federal law and CDC policy (45C.F.R. part 46; 21C.F.R. part 56; 42 U. S⋅C. Sect.241(d); 5 U.S.C Sect. 552a; 44 U.S.C. Sect. 3501 et seq.).

### 2.2. Study period

Tdap vaccination coverage was evaluated retrospectively for pregnancies ending in a live birth in calendar years 2016 through 2023, by year. Some pregnancies ending in live births in 2016 began in 2015; the 2015 enrollment, vaccination, and health care utilization data for those births were included in analyses as described below.

### 2.3. Study population

Each calendar year’s eligible population consisted of women ages 12–55 years with a pregnancy that ended in a live birth of at least 26 weeks’ gestation between January 1 and December 31. Pregnancies were identified using a validated VSD algorithm [15]. The algorithm uses International Classification of Diseases (ICD), and CPT^®^ codes from inpatient, outpatient, and emergency department visits, supplemented with clinical data and is updated weekly to identify ongoing and completed pregnancies.

For inclusion in the analysis, women were required to be continuously enrolled in their participating VSD health system for the duration of their pregnancy to ensure adequate capture of pregnancy information, vaccination, and clinical characteristics. VSD enrollment is defined as health system membership at most sites. At one site enrollment is defined by empanelment within the system, and must include a recent preventive healthcare visit. All pregnancies that ended prior to 26 weeks’ gestation, singleton pregnancies that did not end in a live birth, multiple gestation pregnancies with one or more stillbirths, and pregnancies for which the VSD algorithm could not identify the pregnancy outcome were excluded from analyses. The live birth requirement was in place to avoid potential differential vaccine decision-making and allow for comparison with other data sources.

Women with more than one pregnancy over the study period were included in all respective calendar years’ analyses (i.e., the years of each live birth), as vaccination is recommended in each pregnancy. In the rare instance that a woman had two eligible pregnancies with live births in the same calendar year, both pregnancies were included in that year’s analysis.

### 2.4. Ascertainment of vaccination status

All Tdap vaccines administered in the eligible study population were identified from standardized VSD files using Vaccine Administered (CVX) code 115 [11,12]. The VSD vaccine files include data from the EHR and medical and pharmacy claims, and are supplemented through bidirectional communication with regional or state immunization information systems, with standardized data quality checks and de-duplication of vaccines from multiple sources [16]. Tdap vaccines administered during eligible pregnancies were grouped in analyses by birth year, even if vaccine administration was in the prior calendar year (e.g., a pregnancy with a February 2017 live birth and December 2016 Tdap vaccination would be included in the 2017 analysis).

### 2.5. Analyses

#### 2.5.1. Unadjusted Tdap vaccination coverage by calendar year

For each of the calendar years 2016 through 2023, unadjusted Tdap vaccination coverage was calculated at the pregnancy level by dividing the number of women vaccinated during their pregnancy by the total number of pregnancies identified with live births in that calendar year. For each year, coverage was calculated overall and by maternal age group (12–17, 18–24, 25–34, 35–55 years as of pregnancy start date); maternal race and ethnicity as documented in the EHR using mutually exclusive categories (Hispanic, any race(s); American Indian or Alaska Native, non-Hispanic; Asian, non-Hispanic; Black, non-Hispanic; Middle Eastern or North African, non-Hispanic; Native Hawaiian or other Pacific Islander, non-Hispanic; White, non-Hispanic; another race or multiple races, non-Hispanic; unknown or missing) [17]; and health care utilization (number of medical encounters with a diagnosis during the pregnancy: <10, 10–19, 20–29, ≥30 encounters).

#### 2.5.2. Descriptive analyses among Tdap-vaccinated pregnant women

Among those vaccinated each year, we examined gestational age at Tdap vaccination and receipt of other maternal vaccines during the same pregnancy. Gestational age in weeks was calculated using the pregnancy start date as identified in the electronic medical record, supplemented by gestational age data from the birth certificate where available [15]. Descriptive analyses were limited to receipt of influenza and COVID-19 vaccines. To align with vaccine licensure and recommendation, COVID-19 vaccination was described for calendar years 2021–2023. While first recommended in late 2023, maternal RSV vaccines were not available for most pregnancies in that year’s analysis and were thus excluded.

## 3. Results

Across the 2016 through 2023 study period, the number of eligible pregnancies identified in the VSD ranged from 102,374 to 112,297 each year (Table 1). Twenty-five to 34-year-olds made up the largest age group in each year’s analysis, accounting for 62–63% of pregnancies. Each year, 30–35% of the population was Hispanic and 33–36% was non-Hispanic White; race and ethnicity data were missing for 5–7% of pregnancies each year. In each study year, 60–65% of the population had 10–19 documented health care encounters during the pregnancy. The proportion of pregnancies with <10 encounters increased from 9.0% among those with births in 2019 to 17.5% in 2020, coinciding with a decrease in the proportion with ≥20 encounters (30.2% among those with births in 2019, compared to 20.7% in 2020). The proportion of pregnancies with <10 encounters decreased to 12.8% among those with births in 2023 (Table 1).

Over the study period, overall Tdap vaccination coverage steadily increased pre-pandemic to a peak of 88.7% among women with births in 2019 (versus 84.8% in 2016), thereafter declining among those with births over the next four calendar years to a low of 83.9% among women with births in 2023 (Table 1).

### 3.1.1. Tdap vaccination coverage by demographic characteristics and health care utilization

In all eight calendar years, Tdap vaccination coverage was higher among pregnant women aged 25–34 and 35–55 years, compared to those aged 12–17 or 18–24 years. However, the differences in coverage by maternal age group widened over the study period. In 2016, we observed a 4.6 percentage point difference between the group with the highest coverage (25–34-year-olds; 85.7%) and the group with the lowest coverage (18–24-year-olds; 81.1%). In 2023, the difference between the group with the highest coverage (35–55-year-olds; 85.4%) and the lowest coverage (12–17-year-olds; 74.9%) had grown to 10.5 percentage points. In all age groups, coverage increased from 2016 estimates to peaks in 2019 (2020 among 35–55-year-olds) and subsequently decreased in each calendar year thereafter. However, the amount of decrease in Tdap coverage in later years was variable by maternal age group. From 2019 to 2023, the decrease in coverage among 12–17-year-olds (10.6 percentage points) was more than double that of 25–34-year-olds (5.0 percentage points) and three times that of 35–55-year-olds (3.5 percentage points) (Fig. 1).

Tdap vaccination coverage was highest among non-Hispanic Asian pregnant women in all study years, ranging from 92.0 to 95.7% in each calendar year. Among those with known race and ethnicity, coverage was lowest in all years in pregnancies among non-Hispanic Black women, ranging from 78.0 to 83.6% in each calendar year. The differences in coverage by maternal race and ethnicity also widened over the study period. In 2023, we observed a 15.8 percentage point difference between the group with the highest coverage (non-Hispanic Asian; 93.8%) and the group with the lowest coverage (non-Hispanic Black; 78.0%), an increase from the 12.9 percentage point difference between those groups in 2016. As observed among age groups, Tdap vaccination coverage across race and ethnicity categories increased to peaks in 2019 (2020 among non-Hispanic Asian and Middle Eastern or North African women) with subsequent decreases, aside from an uptick among American Indian and Alaskan Native women in 2023 (Fig. 2). While Tdap vaccination coverage estimates decreased from peak coverage years (2019 or 2020) to births in 2023 among all races and ethnicities, the amount of decrease was variable and ranged from 6.4 percentage points among Hispanic or Latino pregnant women to 1.9 percentage points among non-Hispanic Asian pregnant women.

In each calendar year examined, Tdap vaccination coverage was lowest in pregnancies among women with <10 health care encounters during the pregnancy, compared to those with ten encounters or more. Coverage among women with births in 2023 was 73.6% among those with <10 encounters, compared to 85.9% among those with 20–29 encounters (Fig. 3).

### 3.2. Descriptive analyses of Tdap vaccine recipients

Among the 749,369 total Tdap-vaccinated pregnancies, 94.2% of Tdap vaccines were administered between gestational ages 27 and 36 weeks, coinciding with recommendations. Nearly two thirds (64.9%) of all Tdap vaccines were administered during gestational weeks 27 through 30. The proportion of Tdap vaccines administered at 27 through 30 weeks’ gestation increased in each year of the study period, from 56.0% in 2016 to 70.8% in 2023 (Fig. 4).

Patterns of maternal vaccination among Tdap vaccinated pregnancies are shown in Fig. 5. In calendar years 2016 through 2020, only Tdap and influenza vaccines were recommended during pregnancy. In those years, 66.0–73.5% of Tdap vaccine recipients also received influenza vaccines during their pregnancy. Receipt of influenza vaccines among Tdap recipients decreased to 58.7% among those with births in 2023. COVID-19 vaccines were first recommended in 2021 and 37.9% of Tdap vaccine recipients with births in 2021 also received COVID-19 (with or without influenza) vaccines during their pregnancy. Receipt of COVID-19 vaccines among Tdap recipients decreased to 19.0% among those with births in 2023 (Fig. 5).

## 4. Discussion

This large retrospective analysis of pregnancies within the Vaccine Safety Datalink examined maternal Tdap vaccination coverage among women with births in calendar years 2016 through 2023. In this population of more than 100,000 births each year, overall Tdap vaccination coverage peaked at 88.7% among pregnancies with births in 2019. However, coverage decreased in subsequent years, reversing years of steady gains in coverage pre-pandemic, to a low of 83.9% among women with births in 2023. We identified differences in coverage across demographic characteristics and health care utilization, with the lowest coverage estimates among younger (12–17 and 18–24-year-old) and non-Hispanic Black pregnant women, and those with <10 health care encounters during their pregnancy.

Overall Tdap vaccination coverage estimates within the VSD are higher than other published estimates from the United States from the same period [9], but similar to published estimates in some upper-middle and high income counties [18,19]. Internet panel surveys from CDC reported maternal Tdap coverage estimates from 2019 to 2020 through 2022–2023 and observed lower Tdap vaccination coverage in their survey populations (range 463–836 respondents each year) compared to the estimates within the VSD population. Overall survey coverage estimates ranged from 45.8% in 2021–2022 to 55.4% in 2022–23, compared to the VSD estimates of 85.9% and 83.9% among women with births in 2022 and 2023, respectively [9]. A global systematic review and meta-analysis of maternal pertussis vaccination from 2015 through 2023 in upper-middle- and high-income counties reported higher annual coverage estimates in the United States than the panel surveys [19], but lower overall coverage estimates compared to the VSD. The highest overall United States estimate from the meta-analysis (69.8%, 95% confidence interval 66.3–73.2%) was seen in 2019, in line with findings from the VSD [19]. While point estimates were lower, confidence intervals for 2023 coverage estimates from Brazil, Colombia, El Salvador, Italy, Spain, and Australia overlapped with VSD estimates [19]. The higher Tdap vaccination coverage seen in pregnant women in the VSD population is likely due, in part, to demographic differences in the study populations, such as the overrepresentation of the non-Hispanic Asian population and underrepresentation of the non-Hispanic Black population within the VSD population relative to the United States population. Other contributing factors may include the health system enrollment requirement for the current analysis, the high proportion of the VSD population with private health insurance, and the EHR prompts reminding clinicians to offer recommended vaccines in place at many VSD sites.

While the VSD Tdap vaccination coverage estimates are higher than other United States estimates [9], the VSD saw a decrease in overall coverage among pregnancies with births in later calendar years. From the overall peak in 2019 to the 2023 estimate, annual Tdap vaccination coverage among pregnancies within the VSD decreased 4.8 percentage points (or 5.4% of the 2019 coverage). While the CDC Internet panel surveys found similar coverage among their 2019–20 and 2022–23 survey respondents (53.8% and 55.4%, respectively) [9], the maternal pertussis meta-analysis reported a similar inverted U-shape trend in coverage as the VSD [19]. In the United States, Zhang, et al. reported an increase in coverage from 2015 to 2019, then a − 6.1 annual percentage change from 2020 to 2023 [19]. This pattern was not common across counties included in that analysis, but also present in Argentina, the United Kingdom and Australia, which saw varying increases coverage from 2015 to 2019, followed by varying decreases in coverage from 2020 to 2023 [19]. The decreases in maternal Tdap coverage observed in both the VSD and meta-analysis populations are especially notable in the context of increased reports of pertussis in the United States beginning in 2023 [20,21]. Recent declines in maternal vaccination coverage are not limited to Tdap vaccine. Decreases in maternal influenza vaccination coverage have been reported in the United States and Europe following the COVID-19 pandemic [9,22,23].

As has been reported in other estimates of maternal vaccination coverage in the United States [9,10,23,24], we observed differences in Tdap vaccination coverage by age, race and ethnicity, and health care utilization across the study period. The consistently lower coverage among younger and non-Hispanic Black pregnant women identifies populations that may benefit from focused or additional outreach for maternal Tdap vaccination. Growth in the proportion of the VSD population with <10 health care encounters during pregnancy coincided with the COVID-19 pandemic. Among pregnancies with <10 encounters, 2023 Tdap coverage estimates were 12 percentage points lower than those with ≥10 visits, underscoring the well-established association of prenatal care attendance with vaccine uptake [9,25,26].

We further saw that the differences in Tdap vaccination by age and race and ethnicity widened over the 2016 through 2023 study period. Coverage differences between age groups more than doubled over the study period. Similarly, differences in Tdap coverage estimates between non-Hispanic Asian and non-Hispanic Black pregnant women increased from 2016 to 2023. The widening differences in Tdap vaccination coverage identified here should be corroborated within other pregnant populations.

We observed very high accordance with maternal Tdap recommendations in this population. Most vaccines (94%) were administered within the recommended 27 through 36-week gestational age window, with the proportion of vaccines administered within the earlier part of the 27–36-week window increasing in each calendar year, in line with 2018 updates to the maternal Tdap vaccination recommendations [1]. The robust coverage and alignment with recommendations in this population are notable achievements for the clinicians serving these patients.

While the focus of the current analysis was Tdap vaccination, we examined the uptake of additional vaccines routinely recommended during pregnancy among those who received Tdap. These data, while limited to the 83.9–88.7% of women who accepted Tdap vaccine in each study year, show a change in the pattern of COVID-19 and influenza vaccine uptake during pregnancy between 2021 and 2023. In 2021, the first year all three vaccines were recommended for use in pregnancy, 77% of Tdap vaccine recipients also received COVID-19 vaccine, influenza vaccine, or both during their pregnancy. This proportion decreased in subsequent years, with only 61% of Tdap recipients with births in 2023 receiving ≥1 of the other vaccines while pregnant. The seasonality of COVID-19 and influenza vaccine administration can impact the timing of vaccination relative to pregnancy, but the comparisons across birth years shown here should not be impacted by the timing of vaccine recommendations.

The large population and well-established, comprehensive data infrastructure within VSD provide a robust platform for examining Tdap vaccination coverage over time. Despite these strengths, our study is subject to several limitations. First, the findings are not generalizable to all pregnant women in the United States. While the overall VSD site populations are generally demographically representative of their catchment areas [13,14], it is likely that continuously enrolled, pregnant VSD members may have fewer barriers to care and/or more access to vaccination than the general United States pregnant population. Additionally, our population was limited to pregnancies resulting in a live birth and does not represent Tdap vaccination coverage among pregnancies with non-live birth outcomes. Second, vaccination status may be misclassified in the VSD source data, and coverage may be underestimated if pregnant women receive vaccinations outside the participating delivery systems or the immunization registry’s catchment area. However, missing data on Tdap vaccination outside of the VSD health systems should be minimized by the requirement that all participants have health system enrollment for the duration of their pregnancy, and outside vaccine records have been shown to be well captured by reconciliation practices in place at VSD sites [16]. Third, the small size of some race and ethnicity categories (American Indian or Alaska Native, Native Hawaiian or other Pacific Islander) and age groups (12–17 years) may impact our ability to draw meaningful conclusions in these groups in individual calendar years. However, the overall coverage estimates in these groups and estimated trends over the study period are important data points about these under-studied populations. Finally, we did not collect data on insurance for this analysis and are thus unable to examine differences in Tdap vaccine coverage by insurance type, including Medicaid status, within the VSD population. However, we expect differences may be mitigated by the study’s continuous health system enrollment requirement, which should increase access to care.

Our findings provide valuable data on Tdap vaccination coverage among pregnant women in recent years and include estimates by age group, race and ethnicity, and health care utilization during pregnancy. The decreases in coverage among women with births in later study years are similar to findings of recent decreases in coverage for maternal influenza vaccines and have important public health implications. Continued efforts to promote Tdap vaccination during pregnancy, especially among specific populations with lower vaccination coverage, such as younger and non-Hispanic Black pregnant women, are essential to protect pregnant women and their infants.

## Figures and Tables

**Fig. 1.**
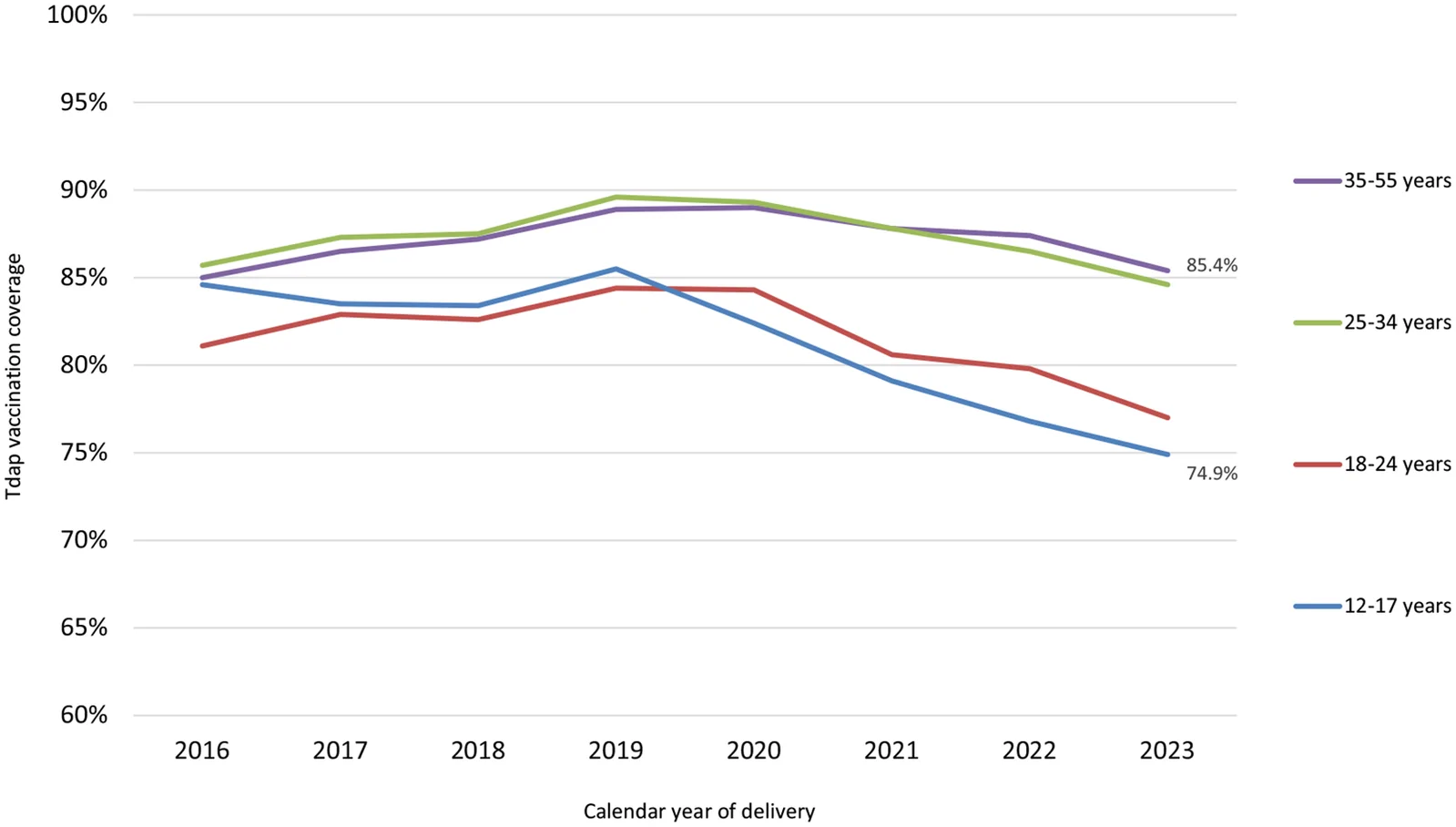
Tdap vaccination coverage among pregnant women ages 12–55 by age group – Vaccine Safety Datalink, 2016 through 2023. Eligible pregnancies were those among women aged 12–55 years at pregnancy start with a live birth of ≥26 weeks’ gestation between January 1, 2016, and December 31, 2023, and continuous VSD enrollment during the pregnancy. All Tdap vaccines administered during the pregnancy were included in coverage estimates. Abbreviations: Tdap, tetanus diphtheria acellular pertussis; VSD, Vaccine Safety Datalink.

**Fig. 2.**
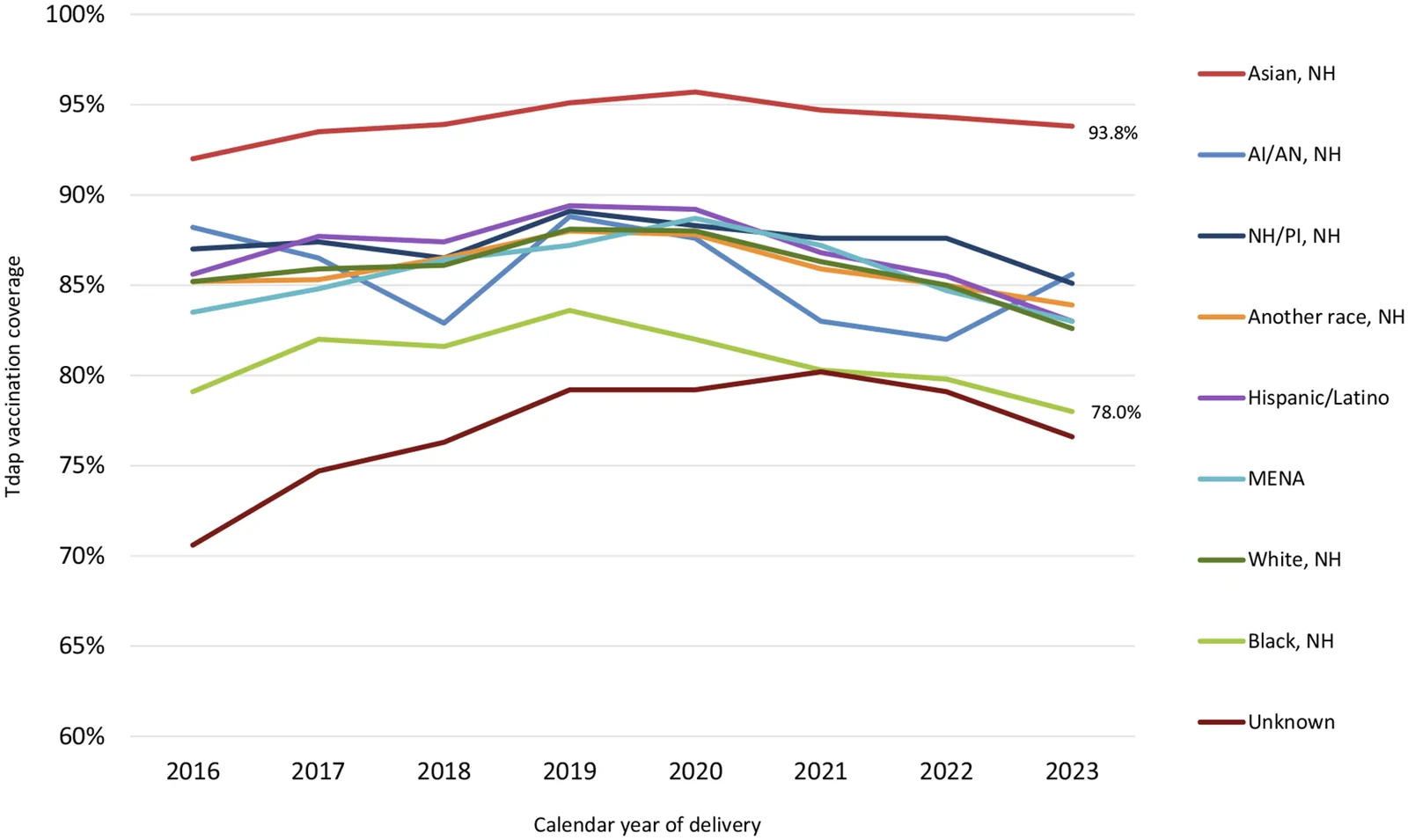
Tdap vaccination coverage among pregnant women ages 12–55 by race and ethnicity – Vaccine Safety Datalink, 2016 through 2023. Eligible pregnancies were those among women aged 12–55 years with a live birth of ≥26 weeks’ gestation between January 1, 2016, and December 31, 2023, and continuous VSD enrollment during the pregnancy. All Tdap vaccines administered during the pregnancy were included in coverage estimates. Race and ethnicity were described as documented in the electronic health record. Abbreviations: Tdap, tetanus diphtheria acellular pertussis; NH, non-Hispanic; AI/AN, American Indian or Alaska Native; NH/PI, Native Hawaiian or other Pacific Islander; MENA, Middle Eastern or North African; VSD, Vaccine Safety Datalink.

**Fig. 3.**
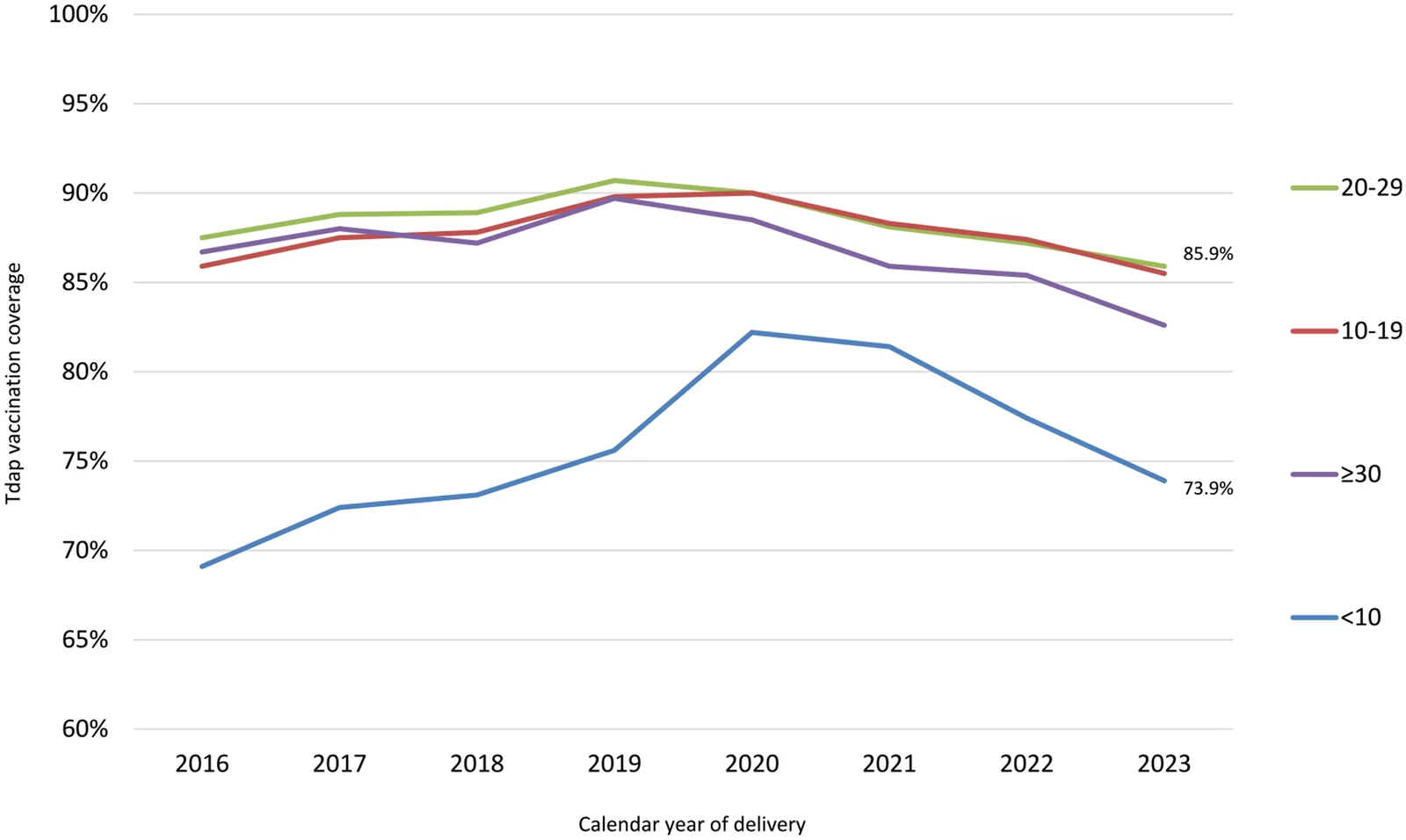
Tdap vaccination coverage among pregnant women ages 12–55 by health care utilization during pregnancy – Vaccine Safety Datalink, 2016 through 2023. Eligible pregnancies were those among women aged 12–55 years with a live birth of ≥26 weeks’ gestation between January 1, 2016, and December 31, 2023, and continuous VSD enrollment during the pregnancy. All Tdap vaccines administered during the pregnancy were included in coverage estimates. Health care utilization was defined as the number of medical encounters with a diagnosis during the pregnancy. Abbreviations: Tdap, tetanus diphtheria acellular pertussis; VSD, Vaccine Safety Datalink.

**Fig. 4.**
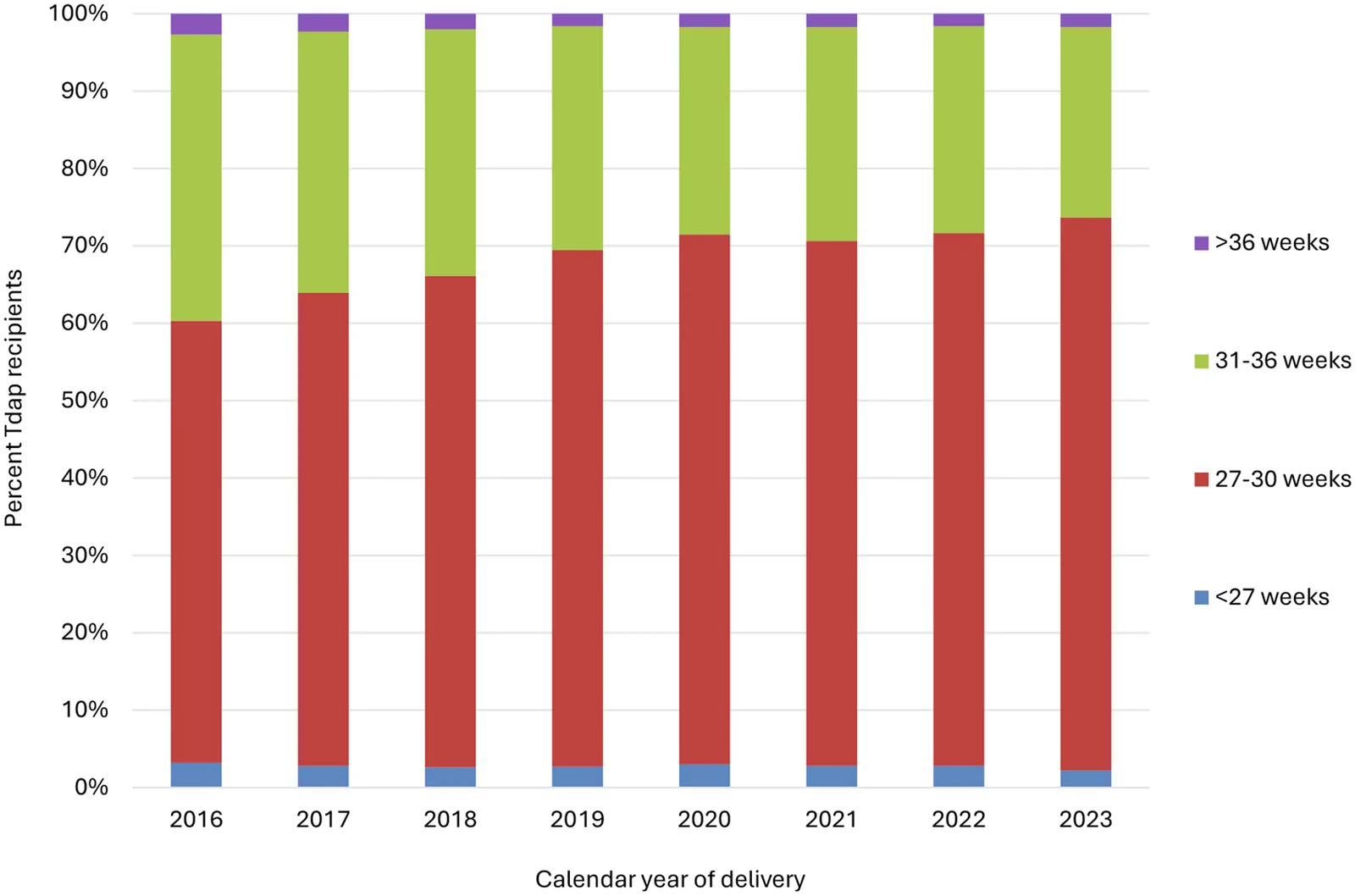
Gestational age at Tdap vaccine receipt – Vaccine Safety Datalink, 2016 through 2023. Eligible pregnancies were those among women aged 12–55 years with a live birth of ≥26 weeks’ gestation between January 1, 2016, and December 31, 2023, and continuous VSD enrollment during the pregnancy. Figure limited to pregnancies with documented Tdap vaccine receipt during the pregnancy. Abbreviations: Tdap, tetanus diphtheria acellular pertussis; VSD, vaccine safety datalink.

**Fig. 5.**
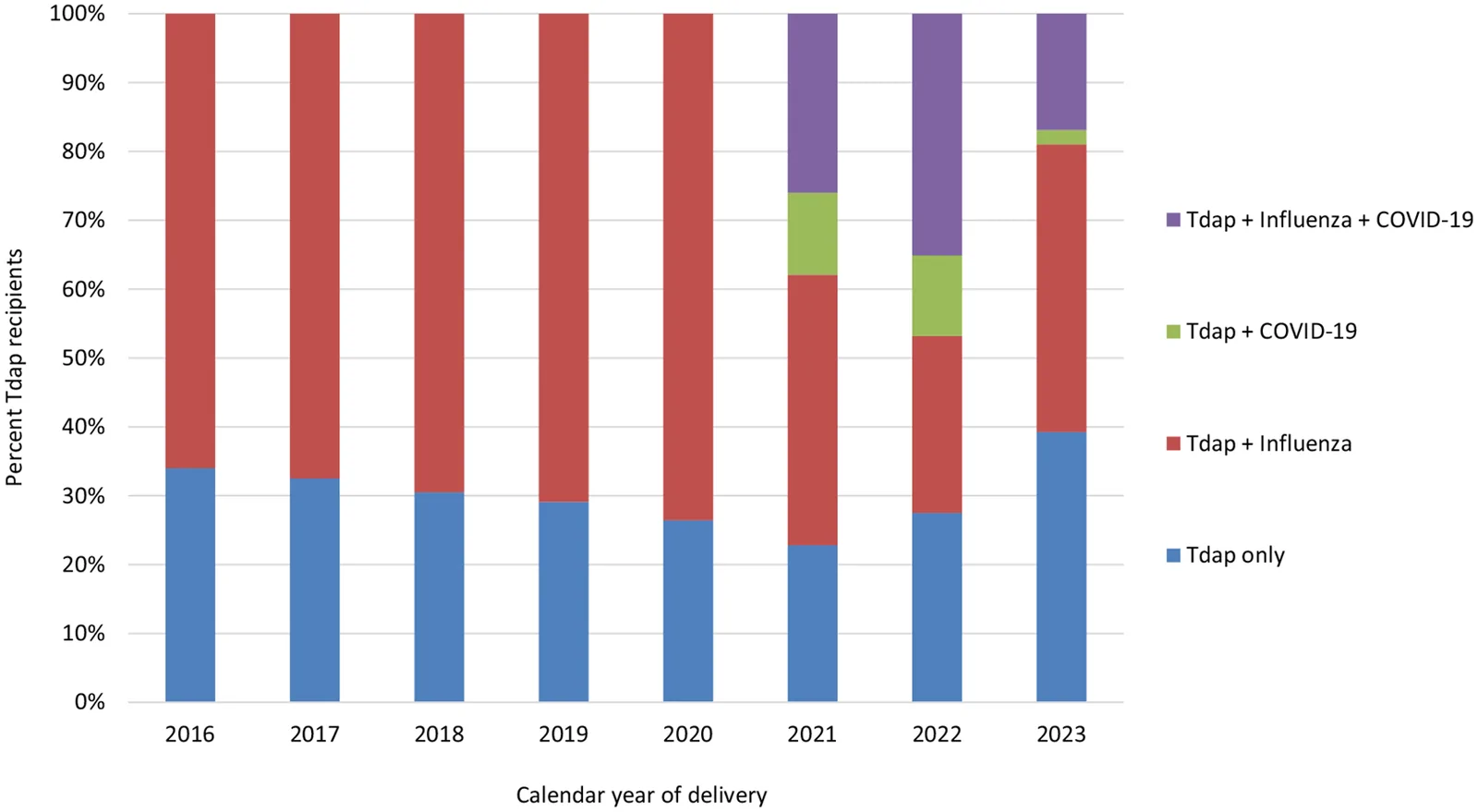
Receipt of influenza andCOVID-19 vaccines during pregnancy among Tdap vaccine recipients – Vaccine Safety Datalink, 2016 through 2023. Eligible pregnancies were those among women aged 12–55 years with a live birth of ≥26 weeks’ gestation between January 1, 2016, and December 31, 2023, and continuous VSD enrollment during the pregnancy. Figure limited to pregnancies with documented Tdap vaccine receipt during the pregnancy. Abbreviations: COVID-19, coronavirus disease of 2019; Tdap, tetanus diphtheria acellular pertussis; VSD, Vaccine Safety Datalink

**Table 1:** Demographic characteristics and Tdap vaccination status of eligible pregnancies in the Vaccine Safety Datalink, 2016 through 2023.

	Calendar year of delivery							
	2016	2017	2018	2019	2020	2021	2022	2023
Pregnancies, n *	103,886	104,449	107,805	109,676	107,333	112,297	109,475	102,374
*Age group, n (col. %)*								
12–17 years	1035 (1.0)	860 (0.8)	764 (0.7)	702 (0.6)	675 (0.6)	569 (0.5)	583 (0.5)	541 (0.5)
18–24 years	16,527 (15.9)	15,750 (15.1)	15,146 (14.0)	14,877 (13.6)	13,776 (12.8)	13,938 (12.4)	12,848 (11.7)	11,802 (11.5)
25–34 years	64,889 (62.5)	65,079 (62.3)	67,621 (62.7)	68,854 (62.8)	67,521 (62.9)	70,874 (63.1)	68,415 (62.5)	63,591 (62.1)
35–55 years	21,435 (20.6)	22,760 (21.8)	24,274 (22.5)	25,243 (23.0)	25,361 (23.6)	26,916 (24.0)	27,629 (25.2)	26,440 (25.8)
*Race and ethnicity, n (col. %)* ^$^								
American Indian or Alaska Native, NH	186 (0.2)	185 (0.2)	205 (0.2)	223 (0.2)	210 (0.2)	230 (0.2)	228 (0.2)	215 (0.2)
Asian, NH	14,637 (14.1)	15,084 (14.4)	15,959 (14.8)	16,815 (15.3)	16,143 (15.0)	16,829 (15.0)	17,017 (15.5)	16,009 (15.6)
Black, NH	7517 (7.2)	7819 (7.5)	8214 (7.6)	8519 (7.8)	8617 (8.0)	9169 (8.2)	8976 (8.2)	8141 (8.0)
Hispanic, any race	31,339 (30.2)	31,761 (30.4)	33,319 (30.9)	34,134 (31.1)	34,661 (32.3)	36,215 (32.2)	37,331 (34.1)	35,946 (35.1)
Middle Eastern or North African, NH	1013 (1.0)	1095 (1.0)	1154 (1.1)	1249 (1.1)	1100 (1.0)	1137 (1.0)	1126 (1.0)	1040 (1.0)
Native Hawaiian or Pacific Islander, NH	407 (0.4)	419 (0.4)	444 (0.4)	451 (0.4)	453 (0.4)	492 (0.4)	510 (0.5)	422 (0.4)
White, NH	37,258 (35.9)	36,776 (35.2)	37,237 (34.5)	37,001 (33.7)	35,431 (33.0)	37,011 (33.0)	33,708 (30.8)	30,569 (29.9)
Other race or multiple races, NH	4193 (4.0)	4270 (4.1)	4522 (4.2)	4759 (4.3)	4612 (4.3)	4949 (4.4)	5077 (4.6)	4745 (4.6)
Unknown/Missing	7336 (7.1)	7040 (6.7)	6751 (6.3)	6525 (5.9)	6106 (5.7)	6265 (5.6)	5502 (5.0)	5287 (5.2)
*Health care utilization, n (col%)* ^								
<10 encounters	9439 (9.1)	9459 (9.1)	9190 (8.5)	9899 (9.0)	18,755 (17.5)	20,989 (18.7)	15,266 (13.9)	13,072 (12.8)
10–19 encounters	62,608 (60.3)	62,807 (60.1)	65,329 (60.6)	66,692 (60.8)	66,352 (61.8)	69,987 (62.3)	70,080 (64.0)	66,769 (65.2)
20–29 encounters	23,845 (23.0)	23,663 (22.7)	24,375 (22.6)	24,100 (22.0)	16,676 (15.5)	15,950 (14.2)	18,006 (16.4)	16,992 (16.6)
≥30 encounters	7994 (7.7)	8520 (8.2)	8911 (8.3)	8985 (8.2)	5550 (5.2)	5371 (4.8)	6123 (5.6)	5541 (5.4)
*Tdap vaccine receipt, n (col. %)*								
Yes	88,094 (84.8)	90,290 (86.4)	93,489 (86.7)	97,266 (88.7)	95,043 (88.5)	97,582 (86.9)	94,025 (85.9)	85,895 (83.9)

Abbreviations: n, number; col., column; NH, non-Hispanic.

* Eligible pregnancies were those among women aged 12–55 years with a live birth of ≥26 weeks’ gestation between January 1, 2016, and December 31, 2023, and continuous VSD enrollment during the pregnancy. In each calendar year a small number of women had more than one eligible birth; this ranged from 22 to 49 women across the study years.

$ Mutually exclusive race and ethnicity categories.

^ Number of medical encounters with a diagnosis during the pregnancy.

## Data Availability

The authors do not have permission to share data.

## References

[1] Liang JL, Tiwari T, Moro P, Prevention of pertussis, tetanus, and diphtheria with vaccines in the United States: recommendations of the advisory committee on immunization practices (ACIP). MMWR Recomm Rep 2018;67(2):1–44. 10.15585/mmwr.rr6702a1. PMC5919600 29702631

[2] Lindley MC, Kahn KE, Bardenheier BH, Vital signs: burden and prevention of influenza and pertussis among pregnant women and infants - United States. MMWR Morb Mortal Wkly Rep 2019;68(40):885–92. 10.15585/mmwr.mm6840e1. PMC6788399 31600186

[3] Centers for Disease Control and Prevention. Provisional pertussis surveillance report. https://www.cdc.gov/pertussis/downloads/pertuss-surv-report-2023_PROVISIONAL.pdf; 2023. Accessed November 11, 2025.

[4] Skoff TH, Hadler S, Hariri S. The epidemiology of nationally reported pertussis in the United States, 2000–2016. Clin Infect Dis 2019;68(10):1634–40. 10.1093/cid/ciy757. 30169627

[5] Havers FP, Moro PL, Hunter P, Hariri S, Bernstein H. Use of tetanus toxoid, reduced diphtheria toxoid, and acellular pertussis vaccines: updated recommendations of the advisory committee on immunization practices — United States, 2019. MMWR Morb Mortal Wkly Rep 2020;69:77–83. 10.15585/mmwr.mm6903a5. PMC7367039 31971933

[6] Sawyer M, Liang JL, Messonnier N, Clark TA. Updated recommendations for use of tetanus toxoid, reduced diphtheria toxoid, and acellular pertussis vaccine (Tdap) in pregnant women – Advisory Committee on Immunization Practices (ACIP), 2012. MMWR Morb Mortal Wkly Rep 2013;62(7):131–5. PMC4604886 23425962

[7] Zhou F, Xu J, Black CL, Trends in Tdap vaccination among privately insured pregnant women in the United States, 2009–2016. Vaccine 2019;37(14):1972–7. 10.1016/j.vaccine.2019.02.042 [Epub 2019 Feb 27]. PMC6557580 30826146

[8] Isenhour CJ, Skoff TH, Lindley MC, Zhou F, Hariri S. Tetanus, diphtheria, and acellular pertussis vaccination coverage among publicly insured pregnant women, U.S., 2016–2019. AJPM Focus 2022;2(1):100060. 10.1016/j.focus.2022.100060. PMC10546573 37789941

[9] Kahn KE, Garacci E, Razzaghi H, Flu, Tdap, and COVID-19 vaccination coverage among pregnant women — United States. https://www.cdc.gov/fluvaxview/coverage-by-season/pregnant-april-2024.html#:~:text=Results,%E2%80%9322%20season%20(45.8%25. [Accessed 18 February 2026]. 10.15585/mmwr.mm7239a4 PMC10545430 37768879

[10] Razzaghi H, Garacci E, Kahn KE, Maternal and infant immunizations for respiratory diseases, United States, May 2025. Vaccine 2026;88:128823. 10.1016/j.vaccine.2026.128823 [Epub 2026 Jun 12]. PMC13366715 42284808

[11] Baggs J, Gee J, Lewis E, The Vaccine Safety Datalink: a model for monitoring immunization safety. Pediatrics 2011;127(Suppl. 1):S45–53. 10.1542/peds.2010-1722H. 21502240

[12] Centers for Disease Control and Prevention. Vaccine safety systems: about the Vaccine Safety Datalink (VSD). https://www.cdc.gov/vaccine-safety-systems/vsd/index.html; 2026. Accessed November 12, 2025.

[13] Sukumaran L, McCarthy NL, Li R, Demographic characteristics of members of the Vaccine Safety Datalink (VSD): a comparison with the United States population. Vaccine 2015;33(36):4446–50. 10.1016/j.vaccine.2015.07.037. Epub 2015 Jul 23. PMC4547875 26209836

[14] Davis AC, Voelkel JL, Remmers CL, Adams JL, McGlynn EA. Comparing Kaiser Permanente members to the general population: implications for generalizability of research. Perm J 2023;27(2):87–98. 10.7812/TPP/22.172. Epub 2023 May 12. PMC10266863 37170584

[15] Naleway AL, Crane B, Irving SA, Vaccine Safety Datalink infrastructure enhancements for evaluating the safety of maternal vaccination. Ther Adv Drug Saf 2021;12:20420986211021233. 10.1177/20420986211021233. PMC8207278 34178302

[16] Groom HC, Crane B, Naleway AL, Monitoring vaccine safety using the Vaccine Safety Datalink: assessing capacity to integrate data from immunization information systems. Vaccine 2022;40(5):752–6. 10.1016/j.vaccine.2021.12.048 PMC8719644 34980508

[17] Williams JTB, Cruz H, Stein A, Breslin K, Brtnikova M, Hepatitis B vaccine series completion by 18 months in infants without a birth dose. JAMA Netw Open 2026;9(4):e269962. /jamanetworkopen.2026.9962. 10.1001/jamanetworkopen.2026.9962 PMC13109789 42030049

[18] Lumbreras Areta M, Valiton A, Diana A, Flu and pertussis vaccination during pregnancy in Geneva during the COVID-19 pandemic: a multicentric, prospective, survey-based study. Vaccine 2022;40(25):3455–60. 10.1016/j.vaccine.2022.04.076 [Epub 2022 May 6]. PMC9076028 35534311

[19] Zhang X, Zhao T, Xie T, Tracking the progress of maternal pertussis vaccination coverage among upper-middle- and high-income countries and territories - a systematic review and modeling study. EClinicalMedicine 2025;90:103651. 10.1016/j.eclinm.2025.103651. PMC12666790 41333898

[20] Centers for Disease Control and Prevention. Pertussis surveillance and trends. https://www.cdc.gov/pertussis/php/surveillance/index.html; 2026. Accessed March 26, 2026.

[21] Centers for Disease Control and Prevention. Pertussis: (Week 52) weekly cases of notifiable disease, United States, U.S. territories, and non-U.S. residents week ending. https://stacks.cdc.gov/view/cdc/175367; December 28, 2024. Accessed March 26, 2026.

[22] López-Zambrano MA, Sánchez-Gómez A, Lasheras Carbajo MD, Trend and factors associated to pertussis and influenza vaccination in pregnant women in Madrid, Spain, 2018–2023 - a retrospective cohort study. Vaccine 2025;68:127895. 10.1016/j.vaccine.2025.127895 [Epub 2025 Oct 28]. 41161042

[23] Irving SA, Crane B, Weintraub E, Influenza vaccination among pregnant people before and during the coronavirus disease 2019 (COVID-19) pandemic. Obstet Gynecol 2023;142(3):636–9. 10.1097/AOG.0000000000005285 [Epub 2023 Aug 3]. PMC10868709 37590982

[24] Irving SA, Crane B, Daley MF, 2023–2024 COVID-19 vaccination coverage in pregnancy in ten healthcare delivery organizations in the United States. Vaccine 2026;79:128441. 10.1016/j.vaccine.2026.128441. Epub 2026 Mar 12. PMC13532371 41825191

[25] Regan AK, Uwimana H, Rowe SL, Systematic review and meta-analysis of interventions to increase the uptake of vaccines recommended during pregnancy. NPJ Vaccines 2025;10(1):76. 10.1038/s41541-025-01120-1. PMC12009365 40253502

[26] Coleman MA, Dongarwar D, Ramirez J, Factors impacting vaccine uptake during pregnancy: a retrospective analysis. Int J MCH AIDS 2022;11(2):e554. 10.21106/ijma.554 PMC9551120 36258713

